# Comparative Genome-Scale Reconstruction of Gapless Metabolic Networks for Present and Ancestral Species

**DOI:** 10.1371/journal.pcbi.1003465

**Published:** 2014-02-06

**Authors:** Esa Pitkänen, Paula Jouhten, Jian Hou, Muhammad Fahad Syed, Peter Blomberg, Jana Kludas, Merja Oja, Liisa Holm, Merja Penttilä, Juho Rousu, Mikko Arvas

**Affiliations:** 1 Department of Computer Science, University of Helsinki, Helsinki, Finland; 2 Department of Medical Genetics, Genome-Scale Biology Research Program, University of Helsinki, Helsinki, Finland; 3 VTT Technical Research Centre of Finland, Espoo, Finland; 4 Department of Information and Computer Science, Aalto University, Espoo, Finland; 5 Institute of Biotechnology & Department of Biosciences, University of Helsinki, Helsinki, Finland; Chalmers University of Technology, Sweden

## Abstract

We introduce a novel computational approach, CoReCo, for comparative metabolic reconstruction and provide genome-scale metabolic network models for 49 important fungal species. Leveraging on the exponential growth in sequenced genome availability, our method reconstructs genome-scale gapless metabolic networks simultaneously for a large number of species by integrating sequence data in a probabilistic framework. High reconstruction accuracy is demonstrated by comparisons to the well-curated *Saccharomyces cerevisiae* consensus model and large-scale knock-out experiments. Our comparative approach is particularly useful in scenarios where the quality of available sequence data is lacking, and when reconstructing evolutionary distant species. Moreover, the reconstructed networks are fully carbon mapped, allowing their use in 13C flux analysis. We demonstrate the functionality and usability of the reconstructed fungal models with computational steady-state biomass production experiment, as these fungi include some of the most important production organisms in industrial biotechnology. In contrast to many existing reconstruction techniques, only minimal manual effort is required before the reconstructed models are usable in flux balance experiments. CoReCo is available at http://esaskar.github.io/CoReCo/.

## Author Summary

Advances in next-generation sequencing technologies are revolutionizing molecular biology. Sequencing-enabled cost-effective characterization of microbial genomes is a particularly exciting development in metabolic engineering. There, considerable effort has been put to reconstructing genome-scale metabolic networks that describe the collection of hundreds to thousands of biochemical reactions available for a microbial cell. These network models are instrumental in understanding microbial metabolism and guiding metabolic engineering efforts to improve biochemical yields. We have developed a novel computational method, CoReCo, which bridges the growing gap between the availability of sequenced genomes and respective reconstructed metabolic networks. The method reconstructs genome-scale metabolic networks simultaneously for related microbial species. It utilizes the available sequencing data from these species to correct for incomplete and missing data. We used the method to reconstruct metabolic networks for a set of 49 fungal species providing the method protein sequence data and a phylogenetic tree describing the evolutionary relationships between the species. We demonstrate the applicability of the method by comparing a metabolic reconstruction of *Saccharomyces cerevisiae* to the manually curated, high-quality consensus network. We also provide an easy-to-use implementation of the method, usable both in single computer and distributed computing environments.

## Introduction

The ability to reconstruct high-quality genome-scale metabolic models is crucial in metabolic modeling and engineering, drug discovery and understanding human disease, such as cancer [1]–[4]. There is a growing gap between the number of sequenced genomes and high-quality, genome-scale metabolic networks stemming from the emergence of high-throughput sequencing and the large amount of manual work needed to curate a metabolic model [5]–[7]. Automatic metabolic reconstruction efforts have so far been hindered by poor-quality sequence data, distant homology, incorrect annotations in biological databases and missing reaction stoichiometry. To match the rate of genome sequencing and to remove an important bottleneck of metabolic analyses, computational methods for metabolic reconstruction must be able to produce models that need only a minimal amount of curation and can still accurately predict metabolic phenotypes [8].

Although metabolic networks have been reconstructed for many microbial species [9]–[12], several industrially important production hosts, such as *Trichoderma reesei*, still lack high-quality genome-scale metabolic networks. Also for other fungal species including major plant pathogens *Ustilago maydis* and *Magnaporthe grisea*, and major human pathogens *Aspergillus fumigatus* and *Candida glabrata*, no curated metabolic models exist. It is thus important to produce metabolic network models for these species that are able to carry out analyses such as flux balance analysis [13].

In this work, we address these challenges by introducing a novel computational method (CoReCo — *Co*mparative *ReCo*nstruction) for simultaneous genome-scale metabolic reconstruction of multiple related species that leverages on the growing availability of sequenced genomes. Importantly, we assume that all species in the phylogeny — present and ancestral — must have a gapless metabolic network, and use this assumption to reconstruct metabolisms that parsimoniously explain the observed sequence data [14], [15]. The ability to automatically produce gapless networks removes an important bottleneck of many existing reconstruction workflows.

We demonstrate the method by reconstructing gapless metabolic models for a large number of fungal species. Although a number of computational approaches to single-species metabolic reconstruction including annotation-based and machine learning strategies have been developed [16]–[22], comparative techniques that exploit the exponentially growing availability of sequence data from multiple related species remain to be developed.

For instance, Model SEED is a software platform for subsystems-based reconstruction and curation of prokaryotic metabolic networks [21]. Similarly to CoReCo, Model SEED annotates complete genomes to reconstruct genome-scale metabolic networks but is limited to single-species reconstruction of prokaryotes. Comparative analyses have been found useful in study of evolutionary histories of fungal transcriptional networks [23] and identification of potential antibiotic targets in metabolic networks [24]. The availability of genomes for many related species enables comparative reconstruction, where sequence data of one species may alleviate issues caused for instance by poor sequencing quality in another. As more genomes are completed and new species are sequenced, comparative reconstruction methods can be rerun to refine existing metabolic models.

Comparative approach to reconstruction offers potential particularly in reconstruction of species that are distant from extensively studied model species, only have an incomplete genome reference sequence available, or when the genome is sequenced at a very low coverage. The latter case is common in for example metagenomics, where a metapopulation of hundreds or thousands of species is sequenced [25].

## Results

### CoReCo: Comparative gapless metabolic reconstruction framework

**Figure 1:**
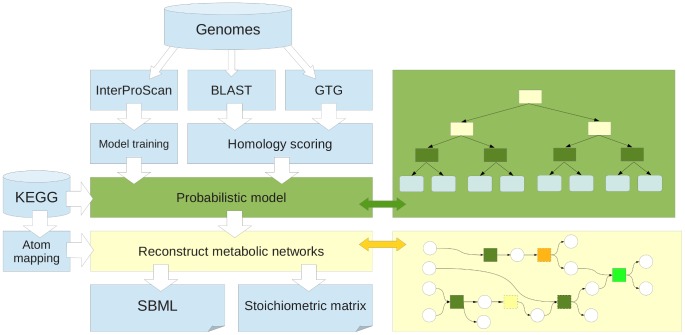
Overview of the reconstruction method. Genomes of target species are subjected to BLAST, GTG and InterProScan analyses to evaluate enzyme probabilities. Plausible gapless metabolic networks are assembled based on integrated enzyme evidence. Finally reconstructed models are converted into SBML and generic stoichiometric matrix formats.

We developed a comparative metabolic reconstruction framework (CoReCo) for simultaneous reconstruction of multiple, related species with a known phylogeny. In addition to a phylogenetic tree, the method requires as input a set of protein coding sequences for each species. The method consists of two phases, illustrated in Figure 1.

In the first phase, sequence data is used to quantify evidence towards existence of known enzymes in each species. To achieve this, the method identifies sequences homologous to input sequences by two distinct techniques, protein BLAST and Global Trace Graph (GTG; [26]). The latter complements BLAST by being able to find more distant homologs. Quantitative results of homolog discovery are integrated in a Bayesian network model, separately instantiated for each enzyme. This results in a posterior probability of each enzyme in each present and hypothetical ancestral species given sequence data. For the second phase, each reaction is assigned the probability of the catalyzing enzyme with highest probability.

Bayesian network topology is derived from the phylogenetic tree. Conditional probability distributions specify the probability of appearance and disappearance of an enzyme at each ancestral species. These species-specific probabilities are estimated from InterProScan enzyme predictions for present species assuming parsimonious phylogeny. Furthermore, conditional probability distributions are estimated for enzyme existence given BLAST and GTG data. Due to the resulting tree topology of the Bayesian network, exact probabilistic inference can be performed efficiently [27].

In CoReCo second phase, a gapless metabolic network is assembled for each present and ancestral species. Utilizing the posterior probabilities computed in the previous phase, our algorithm reconstructs a highly probable network using reactions with low probability only when an addition of a reaction with a high probability would leave the network gapped. The algorithm iterates through reactions meeting with specified probability threshold, and adds the reaction if a gapless biosynthesis pathway producing the reaction substrates can be found. Here, the method takes advantage of precomputed reaction *atom mappings* to accurately find biosynthetic pathways [28]. An additional probability threshold can be used to avoid addition of gapfilling biosynthesis pathways that are not supported well enough by sequence data.

The framework allows efficient parallelization of both phases, thus scaling up to massive datasets. Input protein sequences can be split into arbitrary small sets of sequences to be processed separately by BLAST and GTG. Furthermore, the posterior probability of each enzyme in all species is computed independently of other enzymes. Since the metabolic network for each species is reconstructed separately, also this phase can be parallelized efficiently. In practice, homolog detection with BLAST and GTG is the most time-consuming and also the part of the method where parallelization can be done to an arbitrary degree.

The method produces networks that are gapless in the network connectivity sense: substrates of each reaction in a reconstructed network can be traced to a predefined set of nutrients along reactions in the reconstructed network. Thus networks produced by CoReCo can be utilized with minimal effort in computational analyses requiring structural connectivity such as flux balance analysis. Furthermore, the reactions in the reconstructed models are carbon-mapped, enabling 13C flux analysis [29]. CoReCo produces an Systems Biology Markup Language (SBML) representation for each reconstructed model, annotated with enzyme probabilistic probabilities from phase I as well as carbon mapping for each reaction.

### CoReCo accurately reconstructs poorly sequenced and evolutionary distant species

**Figure 2:**
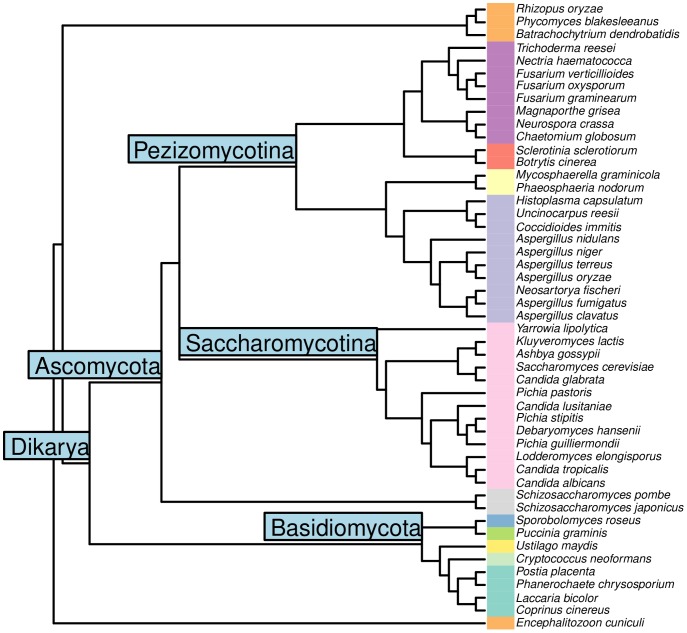
Phylogeny of the 49 fungal species according to [42]. Fungal taxonomic class indicated by colors.

In order to evaluate the usefulness of our method, we comparatively reconstructed 49 fungal species including medically and industrially important species such as *S. cerevisiae*, *T. reesei*, and *P. pastoris* (Figure 2) in two experiments. First, we modified fungal genome data to emulate data from poorly sequenced species and studied the ability of the method to utilize sequence data from related species to recover reconstruction accuracy lost to missing data. Second, we created a scenario which emulated reconstruction of evolutionary distant species. In both settings, sequence data of four *Saccharomycotina* subphylum species *S. cerevisiae*, *K. lactis*, *A. gossypii* and *C. glabrata* were modified and reconstruction performance was evaluated by comparing the reconstructed model for *S. cerevisiae* against a yeast consensus model [30]. The consensus network is a result of extensive collaborative curation efforts, and thus served as the gold standard for our efforts.

Reconstruction accuracy was evaluated in terms of enzyme (EC number) prediction performance. To understand the contribution of individual CoReCo components, we predicted enzyme content also by using BLAST and GTG data separately, as well as a naive Bayesian classifier combining the two. In these classifiers, phylogeny was not used at all. Finally, we performed prediction by CoReCo phase I only, that is, integrating sequence data in the Bayesian network but not doing gapless reconstruction. To compute true and false positive rates for CoReCo phase II, we varied the acceptance threshold parameter. For CoReCo phase I and the naive classifier, different posterior probability thresholds (see Appendix S1) were considered. For BLAST and GTG classification, thresholds for BLAST and GTG scores were used, respectively.

The enzyme content of the resulting *S. cerevisiae* models was compared to the yeast consensus network by counting the number of shared complete EC numbers. A complete EC number specifies all four digits (e.g., 1.1.1.1), whereas an incomplete or partial EC number leaves one or more unspecified (e.g., 1.1.-.-). Our comparison disregarded reactions that had no EC number, such as non-enzymatic or spontaneous reactions. Enzyme-level comparison is invariably inaccurate due to the well-known ambiguity of EC classification. However, we preferred this approach to reaction-level comparison, where the stoichiometries of the two models must be first curated before identical reactions can be accurately identified. A summary of prediction performance in the these settings is given in Table 1.

**Table 1:** Prediction performance of CoReCo phases I and II, naive Bayesian classifier and individual BLAST and GTG classifiers expressed in terms of area under the ROC curve (AUC).

Experiment	BLAST	GTG	Naive	CoReCo I	CoReCo II
Complete data	0.9414	0.8982	0.9652	**0.9668**	0.9455
Poor sequencing	0.8306	0.8209	0.8532	**0.9302**	0.9110
Distant species	0.7289	0.7420	0.7983	0.8600	**0.8825**

Poor sequencing quality was emulated by randomly removing 50% of protein sequences from the four *Saccharomycotina* subphylum species. From this perturbed input data, our reconstruction method was able to reconstruct the *S. cerevisiae* network accurately, displaying clearly increased performance over individual sequence data sources (BLAST, GTG). Accuracy after CoReCo phase I and II was similar. In phase II, reactions that have not been reliably associated with sequences can be added to the network to ensure gaplessness. While producing connected metabolic networks, addition of gapfilling reactions also slightly increases false positive rate of phase II over the phase I result, where gapfilling is yet to be performed. The result demonstrates the benefit of our comparative analysis, where sequence evidence is propagated from neighboring species that are better sequenced and annotated.

We next considered the second setting where the phylogeny includes a small group of species that are closely related to each other but are poorly annotated and have large evolutionary distances from all the other species. In such a scenario, we would expect a large fraction of enzymes in our data to have a low degree of sequence similarity to known enzymes, thus complicating the discovery of correct homologs and functions. To estimate the effect of missing correct annotations would have on reconstruction accuracy, we produced a modified dataset from our fungal dataset by artificially lowering the scores of high scoring sequence matches and comparing the quality of the resulting *S. cerevisiae* reconstruction against the yeast consensus network. Specifically, we modified the BLAST and GTG scores of enzymes in *S. cerevisiae*, *K. lactis*, *A. gossypii* and *C. glabrata* by replacing all BLAST scores over 600 and all GTG scores over 0.99 with scores below these thresholds randomly chosen from *E. cuniculi* data. This process resulted in enzyme scores in the above species closely resembling a typical poorly characterized organism.

**Figure 3:**
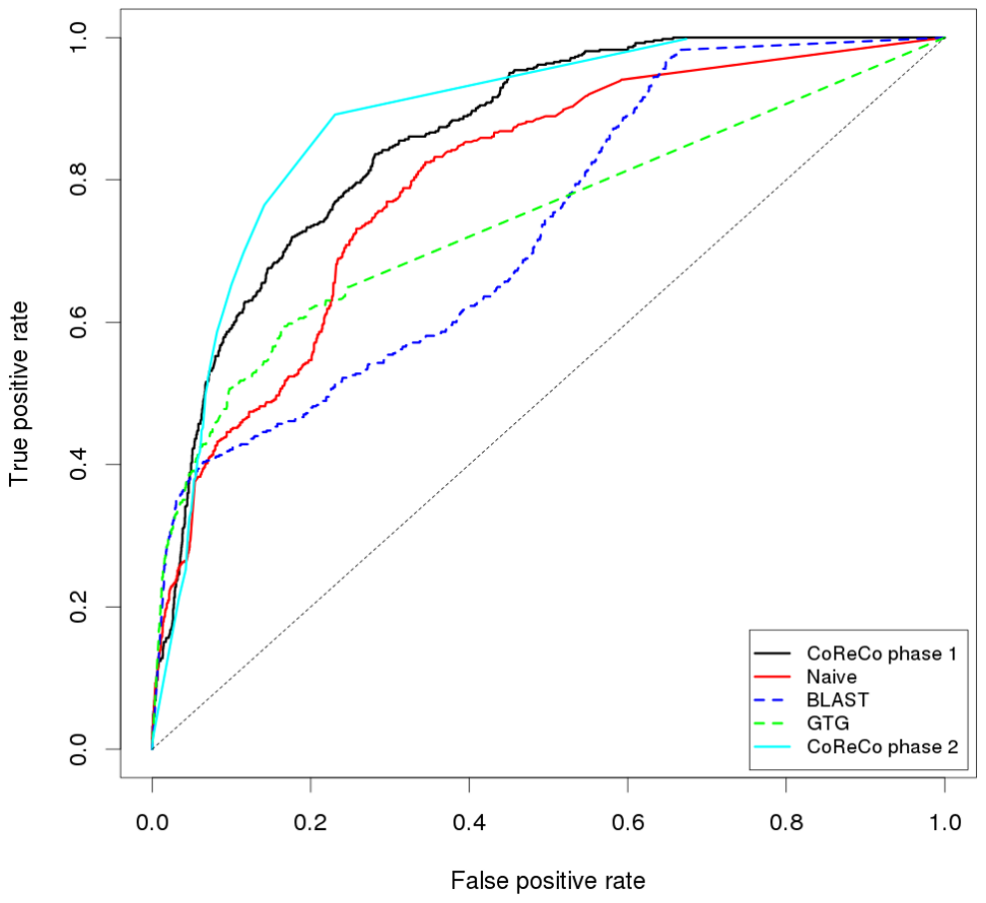
True and false positive rates for *S. cerevisae* consensus model enzyme prediction in the evolutionary distant species setting with the CoReCo phase II reconstructed model (cyan), CoReCo phase I model (black), naive Bayesian classifier (red), and BLAST (blue) and GTG (green) classifiers.

We then computed the true and false positive rates as previously for CoReCo phases I and II, and naive, BLAST and GTG classifiers. Here we observed that our phase II algorithm was able to reconstruct a network much more accurately than the underlying probabilistic model (phase I) alone (Figure 3). This is due to reactions added in gapfilling, which are not predicted by the probablistic model or individual sequence evidence sources. Further, phase I prediction is already markedly better than reconstructing each species independently with the naive Bayesian classifier. These results show that in this setting we clearly benefit from both using a comparative approach (phase I) as well as performing gapfilling (phase II).

### Genome-scale metabolic networks for 49 fungal species

We next proceeded to reconstruct genome-scale metabolic models for the 49 fungal species using unmodified protein sequence data. Reconstruction parameters were chosen values that produced best *S. cerevisiae* CoReCo phase II reconstruction performance in terms of F1 score (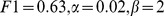) in the above scenario where we reconstructed simulated poorly sequenced species. This process yielded networks also for the 48 hypothetical ancestral species. A minimal amount of manual curation, described in subsequent sections, was applied to the models. Models were converted into SBML format and have been submitted to the BioModels database (BioModels identifiers MODEL1302010000 to MODEL1302010048).

We used whole proteomes of the 49 fungi, a total of 501619 protein sequences, as input to CoReCo phase I. Homologous sequences were retrieved from UniProt and Global Trace Graph databases and scored for each input sequence by BLAST and GTG, respectively. Each enzyme in the KEGG database [31] was assigned BLAST and GTG scores according to best sequence homologs. A posterior probability was computed in the probabilistic model for each enzyme in each species given observed BLAST and GTG scores quantifying our confidence in whether the enzyme is encoded by a particular species.

**Figure 4:**
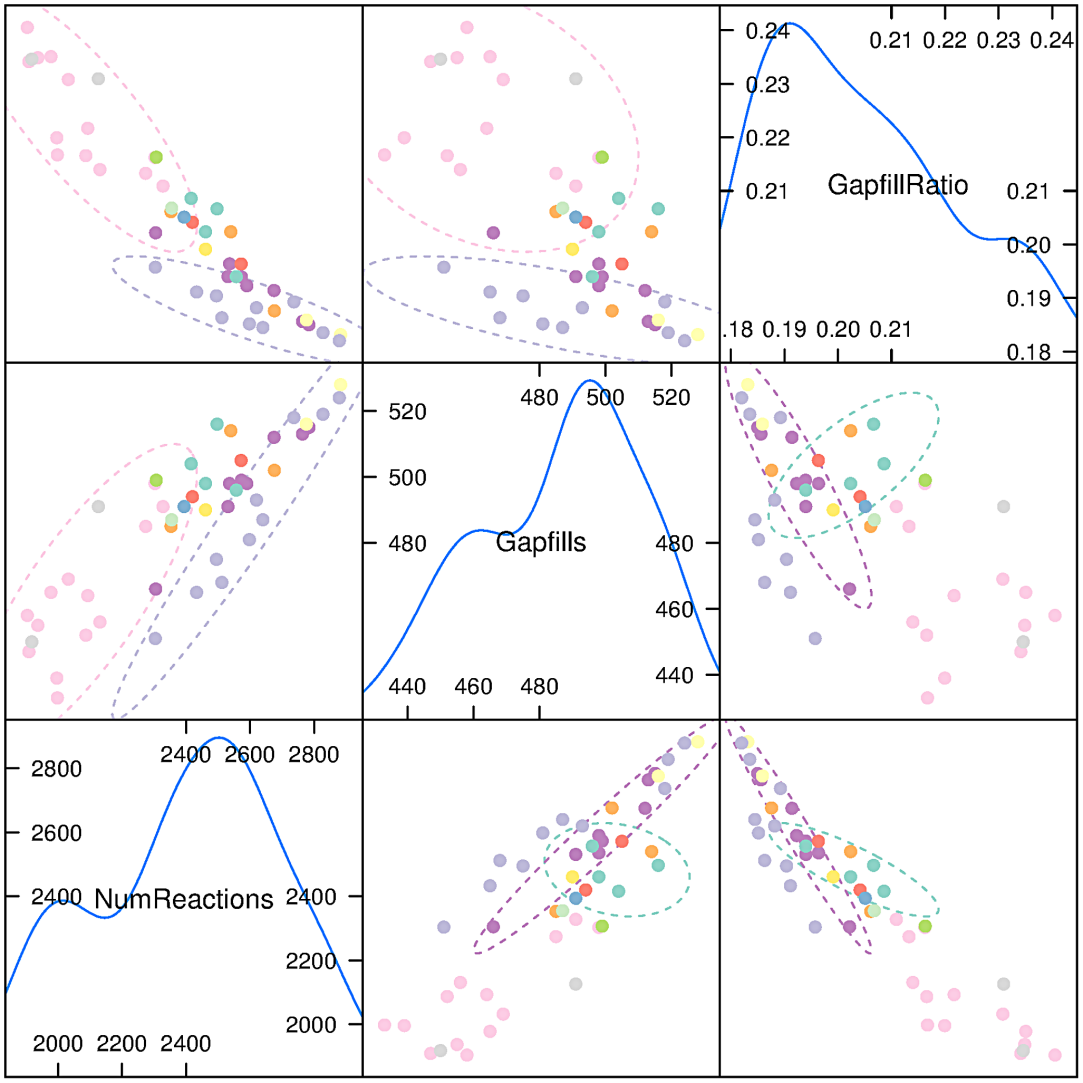
Reconstructed models summarized in terms of number of reactions (NumReactions), number of gapfilling reactions (Gapfills) and fraction of gapfilling reactions to all reactions in the reconstructed model (GapfillRatio). Density plots shown in diagonals. Species colored according to taxonomic class (see Fig. 2). In top left, pink and blue ellipses denote *Saccharomycotina* and *Eurotiomycetes*, respectively. In bottom right, purple and cyan ellipses denote *Sordariomycetes* and *Agaricomycetes*, respectively. Data for *E. cuniculi* not shown.

In CoReCo phase II, metabolic network models were reconstructed based on KEGG stoichiometry data for each present and hypothetical ancestral species. Prior to reconstruction, we balanced 7076 KEGG reactions by allowing addition of protons and water, and a single C1 group. We then computed atom mappings for balanced reactions using a recent algorithm [32]. Atom mapping describes the correspondence of substrate and product atoms in a reaction, and allows removal of biologically spurious pathways [28]. Reconstructed models contained a median of 2215 reactions, including both enzyme-catalyzed and spontaneous reactions, and were associated with a median of unique 1124 enzyme EC numbers. Compared to the enzyme content of the yeast consensus model [30], our reconstructed *S. cerevisiae* model before any manual curation achieved true positive rate of 99.0% and false positive rate of 12.1%. *Saccharomycotina* reconstructions were overall smaller (median of 2032 reactions and 1040 enzymes) than those of other species (Figure 4). As expected, the number of reactions used to fill gaps in reconstructions correlated with model size (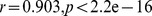). The ratio of gapfilling reactions to all reactions showed negative correlation (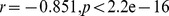), implying that in larger networks a gapped reaction can be fixed with fewer reactions than in smaller networks on the average. Reconstructed *E. cuniculi* model differed from the other models with a total of 737 reactions (341 gapfilling reactions, 46.2% gapfilling ratio). Interestingly, *Eurotiomycetes* models had fewer gapfilling reactions than many species with similar sized models, which probably reflects the fact that many *Aspergillus* species have extensive manual annotation in public databases [33].

### Production of biomass in fungal species

**Figure 5:**
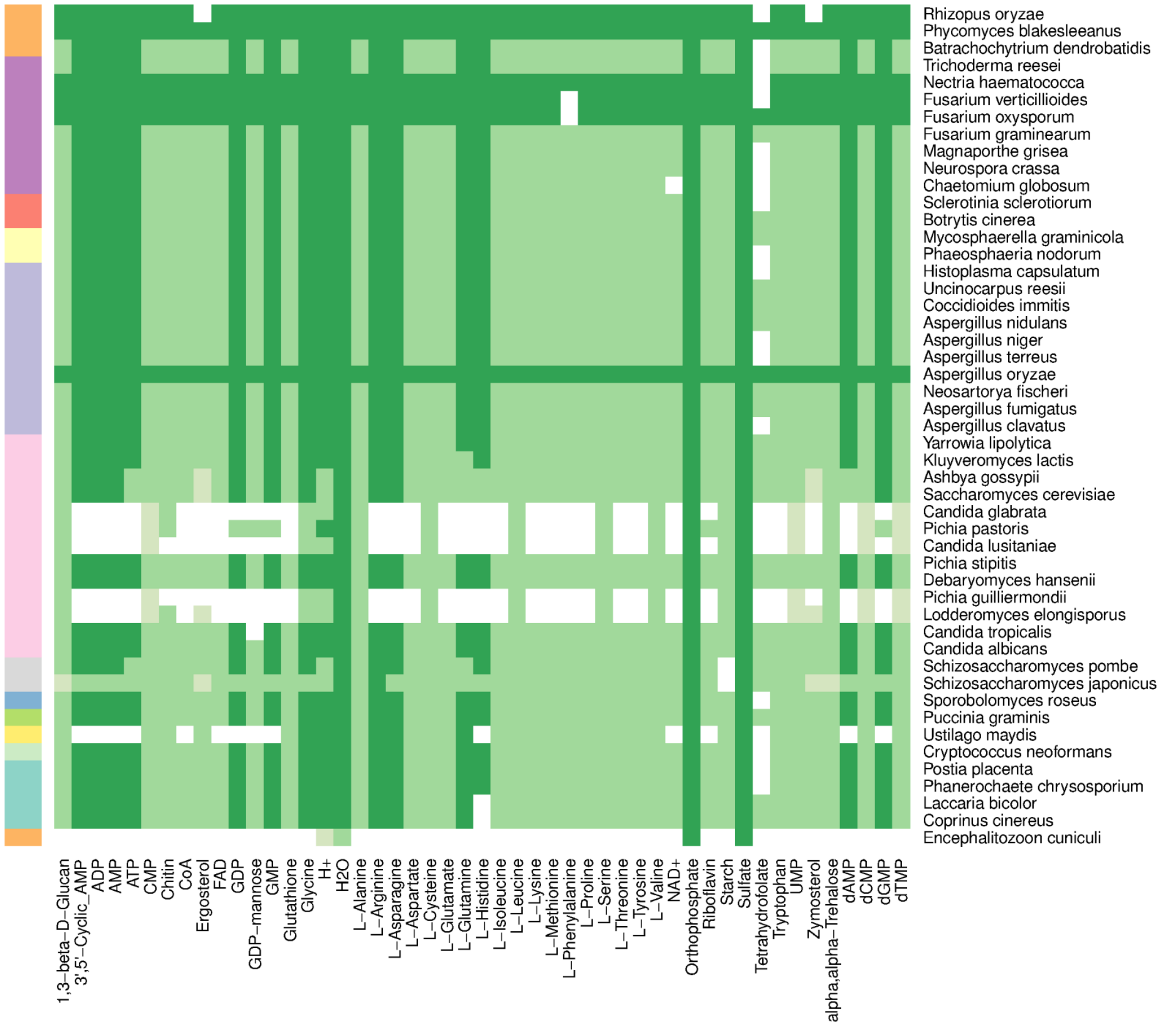
Yields of biomass components in fungal models. Reaction directionality constrained. CO2 assimilation reaction unconstrained. Column-wise normalized yields reported: 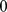 (white), 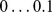 (light green), 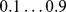 (green), 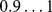 (dark green). Fungal taxonomic class indicated by row colors in left margin.

We next investigated the metabolic capabilities of reconstructed fungal models after minimal manual curation (see Materials and Methods). For each modified model, the maximum yield of biomass components was computed from a minimal media with added bicarbonate under metabolic steady-state. A large fraction of biomass components was producible in most of the fungi (Figure 5). All components and thus biomass could be produced without extensive manual curation in the following 17 species: *A. fumigatus*, *A. gossypii*, *A. nidulans*, *B. cinerea*, *C. albicans*, *C. immitis*, *D. hansenii*, *F. graminearum*, *K. lactis*, *M. graminicola*, *N. fischeri*, *P. blakesleeanus*, *P. graminis*, *P. stipitis*, *S. cerevisiae*, *U. reesii* and *Y. lipolytica*.

For instance, max growth rate in our *S. cerevisiae* model was 0.7388 whereas the growth rate of yeast consensus model on the same media and biomass was 0.7418 [30]. Constraining the reaction directionality proved to be critical, as all biomass components could be produced in 41 of the 49 extant species when no reaction constraints were enforced. Tetrahydrofolate was the least successfully produced component, with zero yield in 25 species. Not surprisingly, only 4 components could be produced in the obligate intracellular parasite *E. cuniculi*. Reconstruction of five *Saccharomycotina* including *P. pastoris* proved difficult and resulted in up to 32 unproducible components. However, in other *Saccharomycotina* such as *K. lactis* and *C. tropicalis* all or nearly all biomass components could be produced.

### Reconstructed fungal models predict growth accurately in knock-out experiments

Lastly we studied the ability of our *S. cerevisiae* model to predict the effects of gene knock-outs on growth. In a recent work, [34] measured growth phenotypes of 465 *S. cerevisiae* gene deletion mutants and compared observations to steady-state model predictions. Here, we compared their results against growth predictions of our final reconstructed *S. cereivisae* model.

We modified the set of 16 growth media used by [34] to take into account KEGG stoichiometry by adding bicarbonate and methyl-THF to each media. Similarly, biomass composition was defined by adjusting the iMM904 biomass composition to reflect metabolite definitions in KEGG. For instance, we replaced glycogen with starch because the former was not involved with any reaction in KEGG. Reaction directionality was constrained as in the biomass production experiment above.

**Figure 6:**
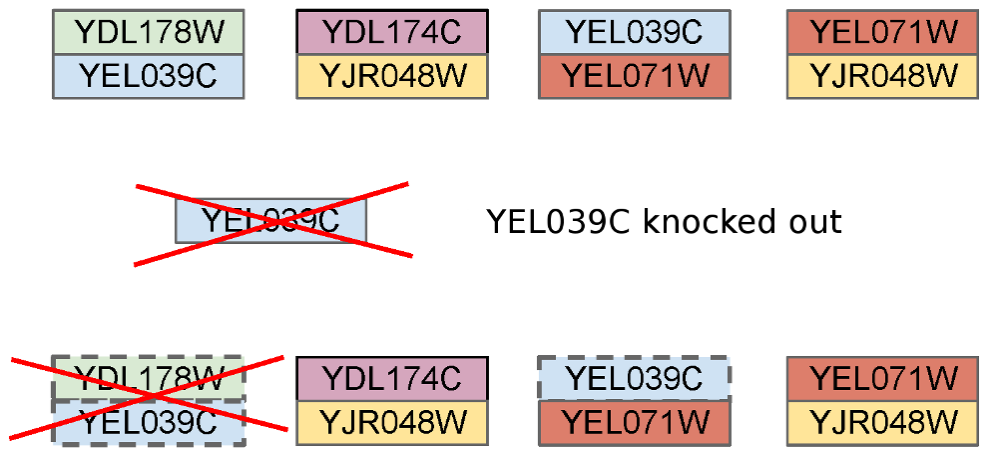
For each *S. cerevisiae* gene knockout, all genes involved in complexes with the deleted gene that do not participate in other complexes are also deleted. For the four complexes shown, YEL039C knockout will result also in the deletion of YDL178W and reactions associated with it because no other YDL178W containing complex remains. In contrast, YEL071W is not deleted, because it participates in a still functional complex with YJR048W.

Gene knockouts in our reconstructed model were simulated according to [34]. For each of the 16 growth media, we performed 465 *in silico* gene knockouts, resulting in 7440 growth prediction cases, and compared our prediction against growth observed in the previous study. To determine the KEGG reactions to remove for each knockout, we mapped *S. cerevisiae* genes to reactions via EC numbers using *Saccharomyces cerevisiae* Genome Database (SGD) and KEGG annotations. A total of 1071 *S. cerevisiae* genes were mapped to 370 EC numbers. We considered the gene associations in protein complexes specified in the yeast consensus model (v4.02) [30] and deleted a reaction only if no functional paralogs annotated with any EC number associated with the reaction remained after the gene knockout (Figure 6). However, in only 116 out of 465 cases (24.9%) at least one reaction was deleted. To remove the effect of trivial positive growth predictions due gene knockout leading to no reaction deletions and thus original yield, we considered only cases where at least one reaction was deleted.

Identically to [34], growth was considered to be successfully predicted if the absolute difference between the normalized model growth rate and the mean experimental growth rate, both ranging from 0 to 1, was less or equal than 0.5. Our model was able to meet the performance of the reference model surprisingly well, achieving 91.6% sensitivity compared to 91.9% sensitivity of the curated reference model (Table 2). In the dataset used, positive cases far outnumber negatives with 1826 positive and 46 negative cases, complicating the estimation of specificity. In conclusion, our computationally generated model was found to be useful in predicting effects of gene knockouts in steady-state conditions despite undergoing only a minimal amount of manual curation.

**Table 2:** Growth prediction accuracy in knockout models with constrained reaction directionality.

Outcome	Observed	Refined	Reference
TP	1673 (89.37%)	1580 (90.60%)	1678 (89.64%)
FP	28 (1.50%)	25 (1.43%)	9 (0.48%)
TN	18 (0.96%)	14 (0.80%)	37 (1.98%)
FN	153 (8.17%)	125 (7.17%)	148 (7.91%)
Total	1872	1744	1872
Sens	0.916	0.927	0.919
Spec	0.391	0.359	0.804
Prec	0.984	0.984	0.995
F1	0.949	0.955	0.955

Reaction deleted only if no functional enzyme complex associated with the reaction remains after knockout. Reconstructed model performance in predicting [34] “raw” observations (Observed) and “refined” observations (Refined) of [34]. Performance of the model of [34] (Reference) in predicting “raw” observations is also shown. Rows: Correct and incorrect growth predictions (TP, FP), correct and incorrect no-growth predictions (TN, FN), total number of cases, sensitivity, specificity, precision and F1 score.

## Discussion

We described a computational method for reconstructing metabolic networks from sequence data for related multiple species and their hypothetical ancestor species. The method is able to exploit shared evolutionary history and alleviate problems caused by lacking sequence data by propagating enzyme evidence from related species. Our reconstruction method allows comparative reconstruction of an arbitrary number of species from sequence and phylogenetic data, and thus can be used to shed light into the evolution of metabolism and predict metabolic phenotypes, for example.

The increasing availability of microbial genomes sequenced individually from cell cultures, or identified in metagenomics experiments has made the rapid and accurate analysis and interpretation of the data the rate-limiting step of biological discovery instead of data acquisition. In metabolic modeling, scaling up metabolic network reconstruction to cope with emerging big sequence data has so far been hindered by the large amount of manual work that goes into curating metabolic models. This problem can be alleviated by enforcing gaplessness in reconstruction [8], [21]. Here we considered metabolic reconstruction as an optimization problem with the goal of producing as plausible gapless model as possible. In our experiments using incomplete sequence data, we observed that such integrated gapfixing resulted in improved reconstruction performance over individual enzyme predictions.

Comparative analysis offers a promising direction for development of metabolic network reconstruction techniques. In this study, we observed significant reconstruction accuracy gains from integration of sequence data from multiple species. Although we used only two distinct sources of sequence information, the Bayesian network framework introduced here can be easily extended to integrate other sequence features as well, or condition- or cell-type specific data [35]. While metabolic models for multiple related species have been reconstructed in previous efforts [24], our framework is to our knowledge the first to exploit sequence data across a known phylogeny to support reconstruction of poorly sequenced and evolutionary distant species. In principle, our method can easily be parallelized to run on a commodity cluster for simultaneous reconstruction of thousands of species.

We discussed here 49 genome-scale metabolic networks of fungal species constructed with our method from protein sequence data. As a proof of concept, we demonstrated high reconstruction accuracy of *S. cerevisiae* network by comparing our results against the high-quality yeast consensus network. Further, our *S. cerevisiae* model was able to accurately predict effects of gene knockouts observed in a previous study [34]. Biological validation of predictions for other fungal species will provide additional insight into the accuracy of the method.

We developed an algorithm for finding plausible gap-filling metabolic pathways extending our previous work [28]. The algorithm exploits atom mapping information [32] to identify metabolic pathways where atom transfer takes place, eliminating many biologically irrelevant metabolic pathways. Despite the computational complexity of the underlying pathway search problem [28], our heuristic algorithm is both fast and achieves good reconstruction accuracy. Approximation techniques may be useful in future work to further improve reconstruction performance.

Evolution of fungal transcriptional networks has been previously studied [23]. Similarly, our approach allows the study of metabolic network evolution from metabolic reconstructions of hypothetical ancestral species. Although we here assume strict vertical evolution, also horizontal gene transfer should be considered in comparative metabolic reconstruction [36]. Another interesting future research direction is automatically determining model compartmentalization [22], [37] to improve accuracy of phenotypic predictions, for example.

## Materials and Methods

### Sequence analysis

CoReCo takes as input the full complement of protein sequences for each reconstructed species. We computed reciprocal alignments of the 501619 protein sequences from the 49 fungi against UniProt Swiss-Prot Release 15.15 sequences with BLAST (blastp 2.2.24+; E-value cutoff 10, default parameters) yielding for each alignment of sequences 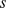 and 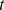 two E-values 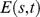 and 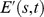 for the forward and reverse BLASTs, respectively. We denote by 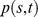 and 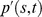 the respective p-values of these E-values. A score describing the joint quality of the two BLAST results for each sequence pair 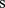 and 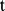 was computed by
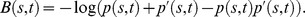
In particular, the score 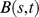 of 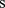 and 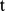 is high only if both p-values are low, capturing the advantages of a reciprocal search to find orthologous sequence candidates [38]. To detect remote homologs, Global Trace Graph (GTG) analysis was performed by first finding the set of conserved amino acid positions 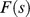 in the GTG alignment for each fungal sequence 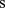. Then, the most similar sequence 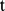 in terms of shared positions, or GTG features, was found for each sequence [26]. Each such sequence pair was given a GTG score by
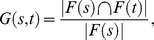
the fraction of shared features.

To evaluate the sequence data support for an enzyme in a single species, we computed a score 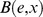 for each enzyme 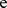 and species 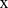 by taking the best BLAST hit annotated with the enzyme,
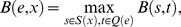
where 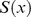 are all protein sequences of the species 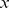 and 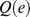 denotes the protein sequences in UniProt annotated with EC number 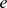. Likewise, we computed GTG scores 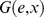 by
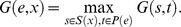



### Reaction stoichiometry and atom mappings

Reactions contained in the KEGG metabolic database (February 2012 version) were filtered by removing all 191 reactions designated as “general reactions”. In KEGG, general reactions do not specify the reactants fully, instead referring to general categories of molecules, such as KEGG reaction R00056 “Dinucleotide+H2O
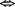
2 Mononucleotide”. We then balanced the remaining reactions by considering the elementwise balances in each reaction formula by integer programming. For each reaction, a balanced formula was found where the coefficient of each existing reactant was at least the original coefficient and at most 15 and the sum of coefficients was minimized. Addition of water and protons, and addition of a single carbonic acid were allowed even if they did not appear in the original formula. Finally, atom mappings were computed for the balanced reactions with a recent algorithm based on A* search and heuristics to prune the search space [32]. Two passes of the algorithm were performed for each reaction. First, an optimal atom mapping was searched for. Whenever the atom mapper was unable to find one within a specified search space limit, a greedy search algorithm was used to find a non-optimal mapping.

### CoReCo phase I: Probabilistic model

**Figure 7:**
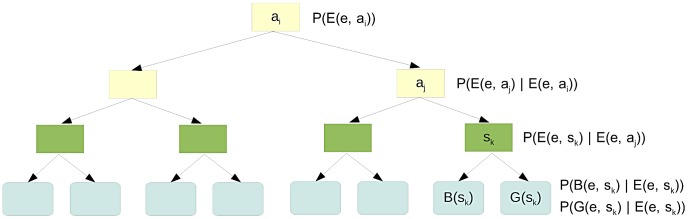
Bayesian network model for computing enzyme probabilities containing three node groups: hypothetical ancestral species (yellow), present species (green), and BLAST and GTG evidence nodes (blue).

We computed species-wise posterior probablities whether an enzyme is encoded by the genome given observed sequence data and phylogeny of the related species. To this end we constructed a Bayesian network model with three groups of nodes (Figure 7). In the model, ancestral and species nodes denote the enzyme in a hypothetical ancestral and present species, respectively. The third group consists of BLAST and GTG evidence nodes for each present species. A separately parametrized instance of the model was constructed for all unique 3006 EC numbers in the data. A conditional probability distribution was specified for each non-root ancestral and present species node by
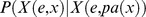
where 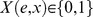 denotes the presence of enzyme 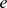 in ancestral or present species 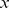 and 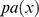 is the parent species of species 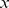. For BLAST and GTG evidence nodes, the conditional probability distribution was given by
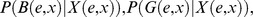
respectively, where 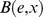 and 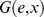 are the BLAST and GTG scores computed for the enzyme 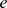 in present species 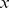. A uniform distribution with 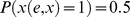 was given to root node to fully specify the model.

To estimate the conditional probability distributions from data, we performed InterProScan (version 1.6) analysis of fungal protein sequences [39]. In contrast to BLAST, InterProScan identifies specific protein family signatures and thus allows us to accurately identify a subset of enzymes present in each species. We included the following 14 InterProScan methods in the analysis: BlastProDom, Coil, FPrintScan, Gene3D, HAMAP, HMMPanther, HMMPfam, HMMPIR, HMMSmart, HMMTigr, PatternScan, ProfileScan, Seg and superfamily. Analysis resulted in 2689551 hits to protein family signatures and a total of 577 unique EC numbers. For instance, 385 unique EC numbers were identified in *S. cerevisiae*.

**Figure 8:**
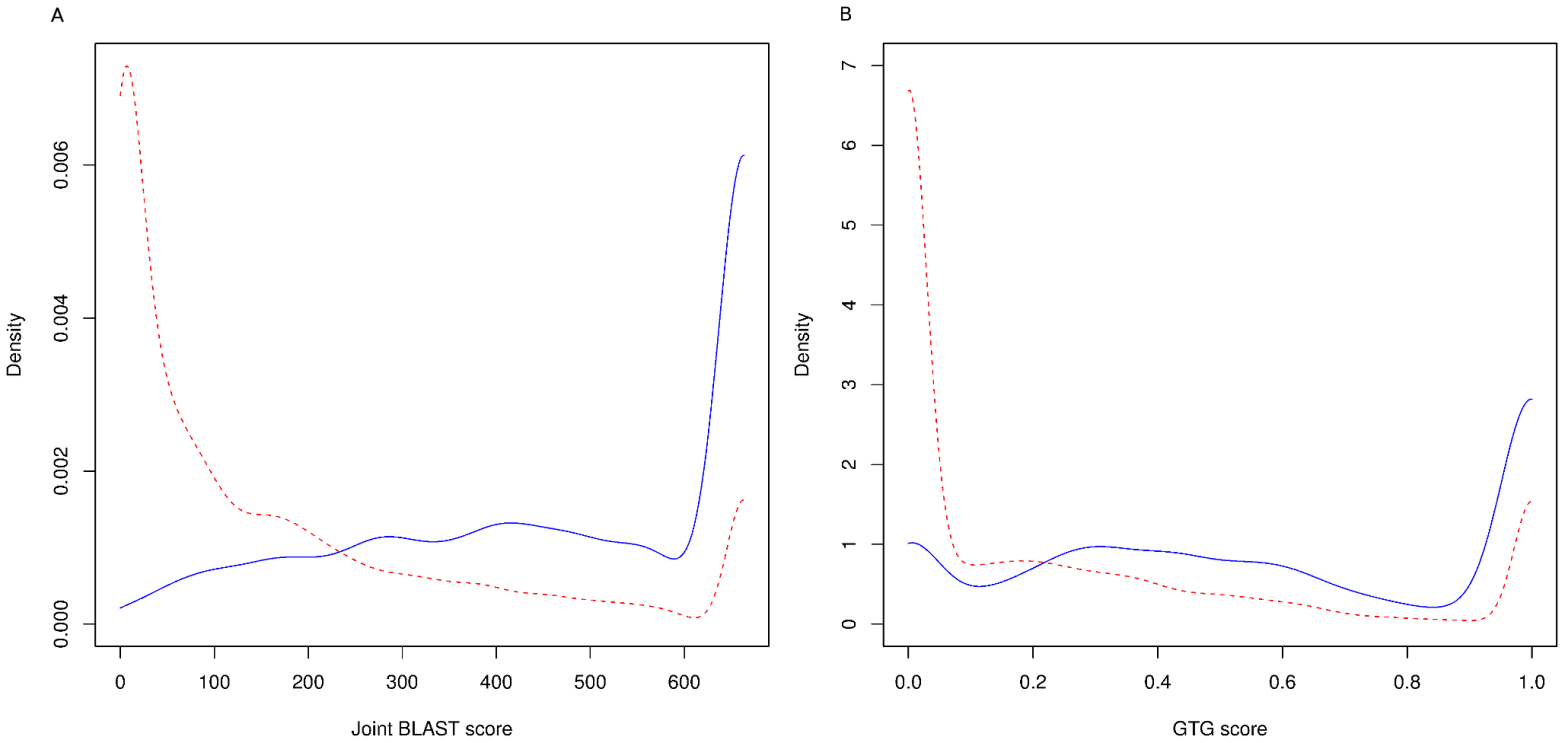
A. Conditional probability distributions for BLAST (left) and B. GTG (right) sequence evidence. Blue solid line: 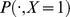, red dashed line: 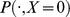.

The EC numbers identified with InterProScan in each species served as reference models when estimating the conditional probability distributions. For evidence nodes, a total of four distributions identical to all species and enzymes were estimated. Probability distribution 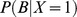 was estimated with a kernel density estimator
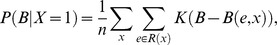
where 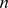 is the total number of summed scores, 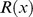 are the enzymes in reference model of species 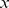, and 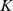 is a kernel function integrating to one. In this study, we used the R function “density” with a Gaussian kernel and estimated the bandwidth parameter with Silverman's method (nrd0). Distributions 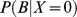, 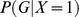 and 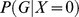 were estimated analogously. To avoid problems with near-zero densities and overfitting, we added 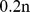 points placed uniformly in range 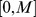 where 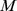 is the maximum score in data for that particular distribution. Figure 8 shows the four estimated conditional distributions.

Conditional probability distributions for species and non-root ancestral nodes were estimated from the InterProScan reference models by first estimating ancestral reference models by parsimony. To do this, we used Fitch's algorithm and estimated ancestral models explaining the reference models such that the number of enzyme additions and deletions were minimized. We then obtained distributions 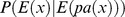 by counting and normalizing the numbers of additions, deletions and no-changes along the edges of the phylogenetic tree.

Finally, we computed the posterior probabilities 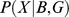 in our model for each enzyme and species with the polytree algorithm [27]. These probabilities served as input to the next phase of our method, where we reconstructed gapless networks for each fungal species.

### CoReCo phase II: Gapless atom-level reconstruction algorithm

To assemble a connected metabolic network from posterior probablities, we developed a novel iterative algorithm, extending our previous work [15], [28], [40]. Our greedy algorithm identifies probable biosynthesis pathways and sequentially adds such pathways into the network being reconstructed. Biosynthesis pathways are constructed by finding a subgraph of an atom graph connecting nutrients to pathway end-products. Here we outline the central operation of the algorithm; details can be found in Appendix S1.

The algorithm assembles each a metabolic network for each individual species 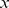 in the dataset independently considering the posterior probabilities 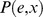 computed previously. Given a database of reactions 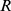, we assign each reaction 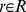 a logarithmic cost
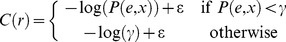
where 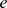 is the enzyme annotated with reaction 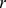 such that posterior probability 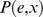 is minimum, 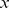 is the reconstructed species and 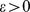 specifies a base cost. In other words, enzyme 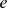 is the enzyme giving maximal support for reaction 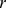. We set 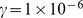 and 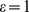 in our experiments. Furthermore, we define the cost of a pathway consisting of reactions 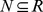 to be simply
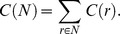



The crucial input parameters to the CoReCo reconstruction algorithm are acceptance threshold 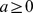 and rejection threshold 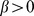. The algorithm attempts to add each reaction whose cost is below threshold 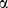 (or equivalently, whose probability is above the corresponding probability threshold) to reconstructed network by finding an inexpensive (in comparison to 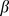) and thus probable biosynthesis pathway leading to the reaction from nutrients. We consider each reaction to transfer each atom from its substrates to products. The correspondence of substrate and product atoms is described by atom mapping of the reaction [32]. In particular, a *complete* atom mapping is a bijection from substrate atoms to product atoms. We require that a valid biosynthesis pathway is able to transfer atoms from nutrients to all metabolites involved on the pathway [28]. The algorithm attempts to satisfy this condition by flagging each atom on the pathway being constructed that is not yet connected to nutrients and considering 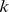 linear reaction paths that connect nutrients to the flagged atoms. In each iteration, a single reaction path is chosen and added to the constructed pathway. This process is repeated until no more flagged atoms remain and the pathway is deemed complete, or the constructed pathway exceeds the maximum cost, i.e., 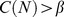. If a complete pathway with cost at most 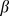 is found, it is added to the reconstructed network.

Heuristics are employed to decrease running time. First, reactions are enumerated in an increasing order of estimated cost of adding the reaction to the network. Addition cost is estimated by finding the shortest reaction path from nutrients and products of already added reactions to each substrate atom of the reaction, and summing the costs of all such paths. Thus reactions that are potentially inexpensive to add to the network are considered first. Addition costs are re-estimated after each iteration, because reaction additions change the cost estimates. Second, the partial pathways are maintained in a priority queue and visited in increasing order of cost estimate for completing the pathway similarly to the first heuristic.

When no complete gapfixing pathways are found for a reaction 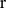 or the best pathway is more expensive than the specified rejection threshold 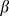, the algorithm can be configured to either reject the reaction 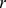 or add it nonetheless. In the first option, we maintain connectivity but cannot predict the reaction even if sequence data solidly supports it. In the second option, we predict the reaction but fail to guarantee connectivity, accepting gaps that rise from addition of sequence-supported reactions. The algorithm flags these reactions in both cases and thus they can serve as focal points for subsequent manual curation.

### Model curation

Following the automatic reconstruction of fungal metabolic models with CoReCo, we manually inspected the central pathways in the reconstructed *S. cerevisiae* model and added reactions that were required for growth and were not included in the reconstruction. These omissions, described in Appendix S1, were due to missing or partial EC numbers in KEGG, and thus were not triggered for gapfilling because sequence evidence could not be connected to these reactions.

### Derivation of reaction direction constraints

Reaction direction constraints for the reconstructed genome-scale metabolic model were derived from Gibbs reaction energy change estimates obtained by the group contribution method [41]. However, formation energy estimates of metabolites required for the calculation of Gibbs reaction energy estimates could not be obtained for compounds lacking a structure formula. Therefore the direction constraints of reactions including reactants lacking a structure formula were manually curated. Additional manual curation was performed to avoid formation of transhydrogenase cycles and to enable the activity of the known central metabolic pathways and biosynthetic pathways of S. cerevisiae. Furthermore, we constrained ATP/O ratio to equal to one. The reaction direction constraints are included in the Table S1.

### Biomass and media definitions

Biomass definition was derived from the iMM904 *S. cerevisiae* model and modified to account for stoichiometry differences between KEGG database and iMM904 model. The minimal media used contained glucose as carbon source Bicarbonate was added to the media, with the restriction that CO2 production would not exceed the intake of bicarbonate. Biomass and media compositions are described in detail in Appendix S1.

## Supporting Information

Appendix S1 Description of the reconstruction algorithm, manual model curation steps, biomass and growth media composition used in experiments, evaluation of the effect of the size of phylogenetic tree to reconstruction accuracy, and posterior probability thresholds used to compute ROC curves.
(PDF)

Table S1 Lower and upper bounds for reaction fluxes used in flux balance analysis experiments.
(TXT)
