# Supplementary material for: Comparative Genome-Scale Reconstruction of Gapless Metabolic Networks for Present and Ancestral Species
Source: PLoS Comput Biol. 2014 Feb 6;10(2):e1003465. doi: 10.1371/journal.pcbi.1003465 (PMC3916221; doi:10.1371/journal.pcbi.1003465)
Supplement: Appendix S1 — Description of the reconstruction algorithm, manual model curation steps, biomass and growth media composition used in experiments, evaluation of the effect of the size of phylogenetic tree to reconstruction accuracy, and posterior probability thresholds used to compute ROC curves. (PDF) [file pcbi.1003465.s001.pdf]

Appendix S1

Comparative genome-scale reconstruction of gapless metabolic networks for present and ancestral species

Esa Pitkänen[1,2,*], Paula Jouhten[3], Jian Hou[1,5], Muhammad Fahad Syed[3], Peter Blomberg[3], Jana Kludas[5], Merja Oja[3], Liisa Holm[4], Merja Penttilä[3], Juho Rousu[5], Mikko Arvas[3]

1 Department of Computer Science, University of Helsinki, 00014 Helsinki, Finland.
2 Department of Medical Genetics, Genome-Scale Biology Research Program, University of Helsinki, 00014 Helsinki, Finland.
3 VTT Technical Research Centre of Finland, 02044 VTT, Espoo, Finland.
4 Institute of Biotechnology & Department of Biosciences, University of Helsinki, 00014 Helsinki, Finland.
5 Department of Information and Computer Science, Aalto University, 00076 Espoo, Finland.
∗ **E-mail: esa.pitkanen@helsinki.fi**

1 Table of Contents

- CoReCo reconstruction algorithm
- Manual model curation
- Biomass definition
- Growth media definition
- Effect of the size of phylogenetic tree to reconstruction accuracy
- Posterior probability thresholds used to compute ROC curves

2 CoReCo reconstruction algorithm

The CoReCo reconstruction algorithm (Algorithm 1) maintains a set of reactions $N \subseteq \mathcal{R}$ representing the reconstructed network. Initially, N is empty. During execution of the algorithm a reaction is added to N either because 1) the reaction is has a high reaction score or 2) it is used to fill a gap. The main loop of the algorithm (lines 4–24) considers each probable, or high-scoring, reaction r in the reaction database $\mathcal{R}$ (such as KEGG) in turn. If the reaction together with the network N reconstructed so far is *complete*, or gapless, the reaction is added to the reconstruction.

To define network completeness, we consider each reaction r to consist of a set of substrate atoms $S(r) \subseteq \mathcal{A}$ and product atoms $P(r) \subseteq A$. An atom mapping M_r defines a bijection between $S(r)$ and $P(r)$. A pathway $N \subseteq \mathcal{R}$ consists of a set of reactions which induce a *atom graph* $G(N) = (U, E)$, where $U = \cup_{r \in N} S(r) \cup P(r)$ and $(u, v) \in E$ if and only if $M_r(u) = v$ for some $r \in N$. Given a set of nutrient atoms $A \subseteq \mathcal{A}$, we say that atom u is reachable from A in N, if and only if there is a (directed) path in $G(N)$ from $a \in A$ to u.

Definition 1 *A pathway N is complete (gapless) if and only if any atom $u \in \cup_{r \in N} S(r) \cup P(r)$ is reachable from nutrients A in N.*

In practice, we often restrict ourselves to the subsets of substrate and product atoms consisting only of carbon atoms. This restriction is useful due to the difficulty of computing high-quality atom mappings for oxygens and nitrogens, for example. Biosynthetic pathways can be often identified despite this restriction, however, as carbon backbone is integral to majority of metabolites [**?**]. For the experiments discussed in the manuscript, we considered only carbon atoms in this regard.

Algorithm 1 Algorithm for assembling a metabolic network

Input: acceptance threshold $\alpha \geq 0$, rejection threshold $\beta > 0$, reaction scores C, $k, n \in \mathbb{N}$, nutrients A
Output: set of reactions $N \subseteq \mathcal{R}$
$N \leftarrow \emptyset$
for all r in $\mathcal{R}$ such that $C(r) \leq \alpha$ **do**
    if $N \cup \{r\}$ is complete **then**
        $N \leftarrow N \cup \{r\}$
    else
        $Q \leftarrow$ `queue`$(\{r\})$ // Priority queue of incomplete pathways
        $P \leftarrow \emptyset$ // Complete pathways
        while $|Q| > 0$ **and** $|P| < n$ **do**
            $q \leftarrow$ `pop`(Q) // Partial pathway with minimum $h(q)$
            $T \leftarrow$ `find_atom_paths`(q, k)
            for all $t \in T$ **do**
                $p \leftarrow q \cup R(t)$ // Candidate pathway p
                if p already visited **or** $C(p) > \beta$ **then**
                    Reject p
                else if p is complete **then**
                    $P \leftarrow P \cup \{p\}$
                else
                    $Q \leftarrow Q \cup \{p\}$
        if $|P| > 0$ **then**
            $N \leftarrow N \cup p$ where $p \in P$ minimizes $C(p)$
        else if accept gaps **then**
            $N \leftarrow N \cup \{r\}$
Return N

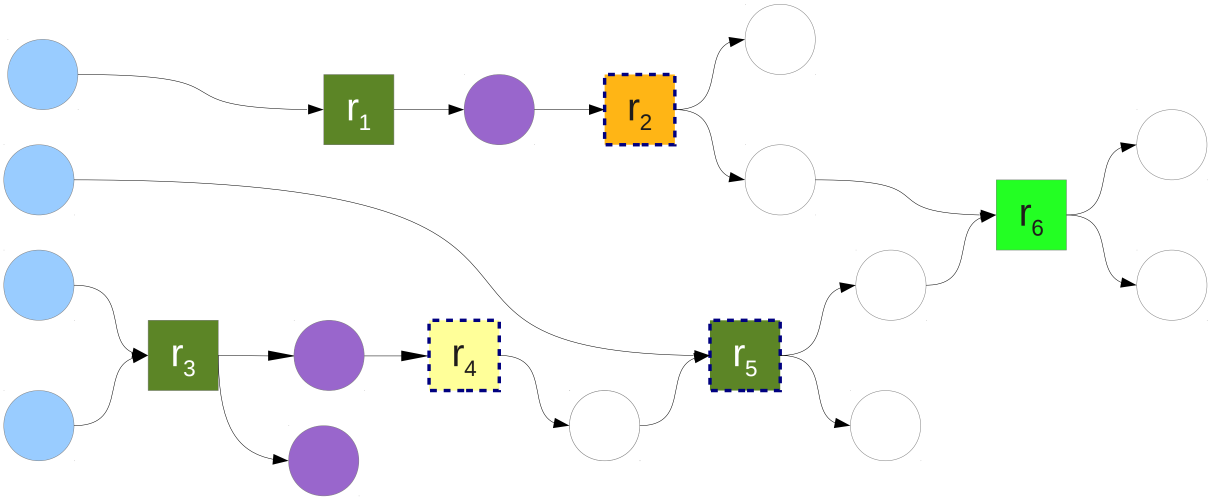

Figure 1. An example of a single iteration of Algorithm 1 on a set of six reactions (rectangles, $r_1, \ldots, r_6$). Metabolites drawn as circles including nutrients (blue) and metabolites reached during previous iterations (magenta). Rectangle colors indicate the degree of evidence towards reactions (green: high, yellow: low). Reactions r_1 and r_3 have already been added to the reconstructed network. To add the highly probable reaction r_6, algorithm finds a biosynthesis pathway for both its substrates. Two iterations of lines 11–20 are required to add the two necessary pathways, first containing reaction r_2 and second reactions r_4 and r_5.

The algorithm attempts to build a gapfilling pathway for each incomplete reaction r (lines 8–24). The search is conducted in reverse from reaction r towards nutrients A. The algorithm maintains a priority queue Q where incomplete, or partial, pathways generated during the algorithm are stored. At each iteration, the partial pathway q with minimum cost estimate $h(q)$ generated so far is augmented to decrease the number of unreached atoms on the pathway. Function h estimates the cost of completing the partial pathway q as the sum of *atom costs* for each unreached atom on the pathway q. The atom cost of atom u is defined as the cost of the minimum cost path in the atom graph $G(\mathcal{R})$ from nutrients A to u. Thus, the cost for adding a pathway to independently produce each unreached atom is considered.

Possible augmentations to the partial pathway are found by computing k shortest atom paths in $G(\mathcal{R})$ from source atoms to atoms of q that are not reachable from nutrients in $G(q)$ (procedure `find_atom_paths`, line 12). In particular, source atoms contain the nutrient atoms A and also the product atoms of reactions already added to the network. For each of these paths, a candidate pathway p is generated (line 14) by adding the reactions $R(t)$ of the atom path t to pathway q. If the candidate pathway is complete, it is added to the list of solution pathways that may be used to gapfill reaction r. Otherwise if the candidate pathway cost does not exceed the rejection threshold β, it is added to the priority queue for possible subsequent augmentation.

When either all candidate pathways or the maximum number of gapfilling pathways per each reaction has been generated, the minimum cost gapfilling pathway is added to the reconstructed network (line 22). If no complete pathway was found, the algorithm may be configured to add the high-scoring reaction to the network nonetheless (parameter 'accept gaps') — this behaviour may result in a gapped reconstruction. It is important to note that connectivity problems in the underlying reaction database leave gaps in reconstructions when plausible reactions cannot be connected to nutrients. In particular, we allowed gaps when we reconstructed models for the 49 fungi presented in the manuscript to include reactions clearly supported by sequence data but for which no gapfixing pathway could be found.

Figure 1 illustrates the operation of the algorithm.

3 Manual model curation

Manual model curation of CoReCo reconstructed models was carried out to ensure positive biomass yield on minimal media in all models. A total of eight reactions were found to be essential to biomass production that were not added by CoReCo into one or more reconstructed models. To rectify this and enable positive biomass yield in all models, these reactions, if missing from a reconstructed model, were added manually. The following table lists the added reactions. Column 'Pathways' shows the KEGG pathways for each reaction. Column 'Comment' shows a reason for omission from models, if any. Two reactions that had a complete EC number were missing from a small number of models. Other reactions had not been associated with protein sequences due to missing or incomplete EC numbers. In addition, one reaction was nonenzymatic. These reactions were missing from the models because they were not used to fill gaps leading to a high-scoring reaction. Column NumModels shows the number of models where the particular reaction was added to.

Reaction	Name	Pathways	Comment	NumModels
R01121	ATP:(R)-5-diphosphomevalonate carboxy-lyase	Terpenoid backbone biosynthesis	Complete EC number	4
R03348	Nicotinate-nucleotide:pyrophosphate phosphoribosyltransferase	Nicotinate and nicotinamide metabolism	Complete EC number	5
R04293	Quinolinate + H2O ⇔ 2-Amino-3-carboxymuconate semialdehyde	Tryptophan metabolism	Nonenzymatic	47
R04457	5-amino-6-(D-ribitylamino)uracil butanedionetransferase	Riboflavin metabolism	No sequence evidence	49
R07505	lathosterol oxidase	Steroid biosynthesis	Incomplete EC number	47
R07506	C-22 sterol desaturase	Steroid biosynthesis	Incomplete EC number	47
R07280	5-Amino-6-(5'-phospho-D-ribitylamino)uracil + H2O ⇔ 5-Amino-6-(1-D-ribitylamino)uracil + Orthophosphate	Riboflavin metabolism	Incomplete EC number	47
R07497	C-8 sterol isomerase	Steroid biosynthesis	No EC number	14

R01121 was added to *A. nidulans*, *E. cuniculi*, *L. elongisporus* and *S. japonicus* models. R03348 was added to *A. nidulans*, *C. globosum*, *E. cuniculi*, *S. japonicus* and *S. pombe* models. R04293 was added to all models except *A. niger* and *L. elongisporus*. R07505 and R07506 were added to all models except *B. cinerea* and *C. globosum*. R07280 was added to all models except *A. gossypii* and *C. glabrata*. R07497 was added to the following 14 models: *A. clavatus*, *B. dendrobatidis*, *C. cinereus*, *E. cuniculi*, *F. graminearum*, *F. oxysporum*, *F. verticillioides*, *H. capsulatum*, *M. grisea*, *P. chrysosporium*, *P. placenta*, *S. cerevisiae*, *S. sclerotiorum* and *T. reesei*.

4 Biomass definition

The following biomass function, derived from the iMM904 *S. cerevisiae* and modified to take into account differences between KEGG and iMM904 model stoichiometry, was used to compute biomass production in steady-state for the 49 fungal models (Table 1).

5 Growth media definition

The following growth media composition was used for computing the steady-state biomass production for reconstructed fungal models (Table 2). The composition is derived from Snitkin *et al.* (2008).

We added 5-Methyltetrahydropteroyltri-L-glutamate uptake because it appears as cofactor in the KEGG reactions 5-Methyltetrahydropteroyltri-L-glutamate:L-homocysteine S-methyltransferase (R04405) and 5-methyltetrahydropteroyltri-L-glutamate:L-selenohomocysteine Se-methyltransferase (R09365), and is a necessary metabolite on cysteine and methionine metabolisms, but no KEGG pathway exists to produce the metabolite from nutrients.

KEGG Compound	Coefficient	Name
C00965	-1.1348	1,3-beta-D-Glucan
C00041	-0.4588	ala-L
C00020	-0.046	amp
C00062	-0.1607	arg-L
C00152	-0.1017	asn-L
C00049	-0.2975	asp-L
C00002	-59.276	atp
C00575	-0.000001	camp
C00461	-0.000001	chitin
C00055	-0.0447	cmp
C00010	-0.000001	CoA
C00097	-0.0066	cys-L
C00360	-0.0036	damp
C00239	-0.0024	dcmp
C00362	-0.0024	dgmp
C00364	-0.0036	dtmp
C01694	-0.0007	ergst
C00016	-0.000001	FAD
C00064	-0.1054	gln-L
C00025	-0.3018	glu-L
C00037	-0.2904	gly
C00369	-0.5185	starch/glycogen
C00144	-0.046	gmp
C00051	-0.000001	gthrd
C00001	-59.276	H_2O
C00135	-0.0663	his-L
C00407	-0.1927	ile-L
C00123	-0.2964	leu-L
C00047	-0.2862	lys-L
C00073	-0.0507	met-L
C00003	-0.000001	NAD
C00079	-0.1339	phe-L
C00148	-0.1647	pro-L
C00255	-0.00099	ribflv
C00065	-0.1854	ser-L
C00059	-0.02	SO_4
C00101	-0.000001	thf
C00188	-0.1914	thr-L
C01083	-0.0234	tre
C00078	-0.0284	trp-L
C00082	-0.102	tyr-L
C00105	-0.0599	ump
C00183	-0.2646	val-L
C05437	-0.0015	zymst
C00096	-0.8079	GDP-mannose
C00008	59.276	ADP
C00080	117.40002	H
C00009	59.305	Orthophosphate
C00035	0.8079	GDP

Table 1. Biomass composition used in experiments.

Name	KEGG compound	Coefficient
Water	C00001	1000.0
Glucose	C00031	22.6
Bicarbonate	C00288	100.0
Diphosphate	C00013	100.0
Iron	C00023	100.0
Oxygen	C00007	6.3
NH3	C00014	100
SLF	C00059	100
PI	C00009	0.89
Potassium	C00238	4.44
Sodium	C01330	0.75
Biotin	C00120	0.00000142
Choline	C00114	0.000092
Inositol	C00137	0.00193
(R)-Pantothenate	C00864	0.0002
Uracile	C00106	0.4
Antimycin	C11339	1000
5-Methyltetrahydropteroyltri-L-glutamate	C04489	12000

Table 2. Growth media used in experiments.

6 Effect of the size of phylogenetic tree to reconstruction accuracy

We evaluated the effect of varying the number of related species to reconstruction accuracy. At the same time, we also varied the fraction of protein sequences of *S. cerevisiae* randomly deleted. Seven subtrees of the original phylogenetic tree of 49 fungi with *S. cerevisiae* and $n \in \{1, 2, 5, 10, 20, 30, 40\}$ related species (Figure 2) along with the original tree of 49 species were constructed. Deletion frequency was varied from 0 to 0.5 in increments of 0.1. A metabolic network *S. cerevisiae* was then reconstructed given each combination of a phylogenetic tree and deletion frequency, and the result compared against the Yeast consensus model [?]. We summarized the reconstruction accuracy as Area Under Curve of the ROC curve shown in Figure 3.

In an unperturbed case with no deleted sequences, we observe that the reconstruction accuracy is already at a high level with 5 neighbors in the phylogenetic tree, reaching maximum with 20 neighbors. When protein sequences are removed from yeast, the reconstruction quality drops somewhat as expected, with local optimum with 5 neighbors. The results suggest that when dealing with comprehensive sequence data and well-annotated related species, only a few related species are needed for a high-quality reconstruction. However, in our experiment, having only a small number of related species gives an edge over a larger number likely due to the noise introduced by the additional species further away from *S. cerevisiae*.

7 Posterior probability thresholds used to compute ROC curves

The following probability thresholds α were used to compute ROC curves shown in Figure 3 of the manuscript:

0, 0.0000001, 0.000001, 0.00001, 0.0001, 0.001, 0.005, 0.01, 0.015, 0.02, 0.025, 0.03, 0.035, 0.04, 0.05, 0.06, 0.07, 0.08, 0.09, 0.1, 0.15, 0.2, 0.25, 0.3, 0.35, 0.4, 0.45, 0.5, 0.55, 0.6, 0.65, 0.7, 0.75, 0.8, 0.85, 0.9, 0.95, 0.99, 0.999, 0.9999, 0.99999, 0.999999, 1.

!h

figs/jiansfig/S_tree_1.png

figs/jiansfig/S_tree_20.png

figs/jiansfig/S_tree_2.png

figs/jiansfig/S_tree_5.png

figs/jiansfig/S_tree_30.png

figs/jiansfig/S_tree_10.png

figs/jiansfig/S_tree_40.png

Figure 3. *S. cerevisiae* reconstruction accuracy compared to Yeast consensus model with respect to varying the number of species related to *S. cerevisiae* specified in the phylogenetic tree (neighbors, $n \in \{1, 2, 5, 10, 20, 30, 40, 48\}$) and fraction of deleted protein sequences of *S. cerevisiae* given as Area Under Curve (AUC).
